# A new species of *Solanum* named for Jeanne Baret, an overlooked contributor to the history of botany

**DOI:** 10.3897/phytokeys.8.2101

**Published:** 2012-01-03

**Authors:** Eric. J. Tepe, Glynis Ridley, Lynn Bohs

**Affiliations:** 1 Department of Biology, University of Utah, 257 South 1400 East, Salt Lake City, Utah 84112, USA; 2 Department of Biological Sciences, University of Cincinnati, 614 Rieveschl Hall, Cincinnati, Ohio 45221, USA; 3 Department of English, University of Louisville, 2211 South Brook, Louisville, Kentucky 40292, USA

**Keywords:** Amotape-Huancabamba zone, Andes, Ecuador, Jeanne Baret, new species, Peru, *Solanum* section *Anarrhichomenum*

## Abstract

We describe *Solanum baretiae* **sp. nov.**, a new species of *Solanum* section *Anarrhichomenum*, named in honor of Jeanne Baret, who sailed as the assistant to botanist Philibert Commerson on Louis Antoine de Bougainville’s global circumnavigation (1766–1769). The species is similar to *Solanum chimborazense*, but differs in having larger flowers, more flowers per inflorescence, and different patterns of pubescence on the filaments (pubescent adaxially and glabrous abaxially) and style (papillose to sparsely pubescent). A description, illustration, photos, and comparisons to similar species are included. Also included is a preliminary conservation assessment, along with a brief account of the important role played by Baret during the expedition. The new species appears to be restricted to the Amotape-Huancabamba zone, an area of southern Ecuador and northern Peru known for its exceptional biodiversity.

## Abstract (es)

Se describe *Solanum baretiae* **sp. nov.**, una nueva especie de *Solanum* sección *Anarrhichomenum*, en homenaje a Jeanne Baret, quien viajó como asistente del botánico Philibert Commerson en la circunnavegación mundial de Louis Antoine de Bougainville (1766–1769). La especie se asemeja a *Solanum chimborazense* pero se diferencia de ella por sus flores más grandes, más flores por inflorescencia, y por tener patrones diferentes de pubescencia en los filamentos (pubescentes adaxialmente y glabros abaxialmemte) y el estilo (papiloso a escasamente pubescente). Se presentan una descripción, ilustración, fotos, y comparaciones con especies similares. Se incluye también una estimación preliminar de su conservación, junto con una descripción breve del papel importante que tuvo Baret durante la expedición. La especie nueva parece estar restringida a la zona Amotape-Huancabamba, un área del sur de Ecuador y norte de Perú notable por su excepcional biodiversidad.

## Introduction

Botanizing in the 15^th^–19^th^ centuries, when naturalists traveled on board ships that sailed to little known parts of the world, must have been truly extraordinary. The hardships endured on these voyages are unfathomable to field biologists these days and, although many of the plant families seen on voyages across the seas were familiar to European naturalists, many of the genera and nearly all of the species encountered and collected were new to Western science. Discovery on such a grand scale is no longer a reality, but detailed focus on groups of plants reveals that a great deal of diversity remains to be uncovered. Fieldwork associated with the PBI (Planetary Biodiversity Inventory) *Solanum* project has resulted in the collection or description of nearly 50 new species of *Solanum* L.to date (recent examples include Tepe and Bohs 2009, Stern and Bohs 2009, 2010, Farruggia and Bohs 2010, Farruggia et al. 2010, Knapp 2010a, b, Vorontsova and Mbago 2010, Vorontsova et al. 2010), including the new species of *Solanum* sect. *Anarrhichomenum* Bitter from southern Ecuador and northern Peru described here.

*Solanum*, with an estimated 1500 species, is not only one of the world’s largest genera of plants (Frodin 2004), but, considering that it includes the tomato (*Solanum lycopersicum* L.), potato (*Solanum tuberosum* L.), and eggplant (*Solanum melongena* L.), it is also one of the most economically important. The PBI: *Solanum* project is an effort to provide a worldwide revision of *Solanum* and make data freely available online at www.solanaceaesource.org; a taxonomic revision of *Solanum* section *Anarrhichomenum* forms part of this project. The section encompasses a group of 10 to 20 viny species found primarily in mountainous habitats from Mexico to Bolivia (Correll 1962, Nee et al. 2006). It is closely related to the pepino (*Solanum muricatum* Ait.) and the clade that contains the tomato and potato (Spooner et al. 1993), and is part of the larger Potato clade *sensu* Bohs (2005) and Weese and Bohs (2007). Members of this section can be distinguished by fruits that mature to red or orange, seeds with a prominent wing in most species, and the presence of a single or strongly anisophyllous pair of pseudostipules at each node. Pseudostipules, which are leaf-like, often crescent-shaped appendages located near the point of petiole insertion, are present in several groups within *Solanum* and other genera of Solanaceae. They do not appear to be part of the leaves that they accompany, but are instead interpreted to be the first leaf or leaves of an arrested axillary shoot (for further discussion see Spooner et al. 2004, Peralta et al. 2008). The leaves of many species are also punctate with whitish deposits of crystal sand (“sand punctate” hereafter). Cells containing deposits like these are found in several groups within *Solanum* (Whalen et al. 1986, Bohs 1990, Knapp 1992) and in other groups of angiosperms (Metcalfe and Chalk 1983).

Correll (1962) provided a revision of *Solanum* section *Anarrhichomenum* [as *Solanum* section *Tuberarium* (Dunal) Bitter subsection *Basarthrum* Bitter series *Appendiculata* Rydb.] in his monograph of the potatoes *Solanuml*. Subsequent studies have clarified the limits of the section as well as many of its component species using a variety of techniques, including morphological examination of the plants, pubescence, pollen, and chromosomes (Anderson 1979a, 1979b, Anderson and Gensel 1976, Anderson and Levine 1982, Seithe and Anderson 1982, Levine and Anderson 1986, Bernardello and Anderson 1990, Anderson et al. 1999), biosystematic studies (Mione and Anderson 1992), analyses of foliar flavonoids (Anderson et al. 1987), and, most recently, molecular techniques (Spooner et al. 1993, Anderson and Jansen 1994). Despite this attention, other species of *Solanum* section *Anarrhichomenum* remain poorly understood and new species exist. The section is currently under revision by the first author, who is attempting to update Correll’s (1962) treatment. This study will incorporate results from the studies mentioned above, specimens from extensive collecting in recent decades, and data derived from additional morphological and molecular studies. During this work, the following new species was recognized.

Throughout this paper, herbarium barcodes and accession numbers are listed in brackets; barcode numbers include the herbarium acronym within the brackets, whereas accession numbers are listed as the number only, without the acronym.

## Taxonomic treatment

### Solanum baretiae

Tepe sp. nov.

urn:lsid:ipni.org:names:77116659-1

http://species-id.net/wiki/Solanum_baretiae

Figs 1 –2 

#### Diagnosis.

Solano chimborazensi *Bitter primo adspectu maxime similis sed floribus maioribus et pilis e filamentis abaxialiter plerumque carentibus differt.*

#### Type.

PERU: Cajamarca: Prov. Contumazá, Bosque de Cachil, 2500 m, 28 Jun 1992 (fl), *A. Sagástegui A. et al. 14710* (holotype: HUT [028009] (photo); isotypes: F [2114228] (photo), GB [0167885], NY [NY00726434]).

#### Description.

Vine, trailing along ground or climbing on other vegetation to 3 m or more, rooting at the nodes. Stems slender, woody, moderately to densely covered with crisped transparent to tawny pubescence of unbranched, eglandular, multicellular trichomes. Sympodial units plurifoliate, not geminate. Leaves simple to 7-pinnate, most commonly 3–5-pinnate, the blades 0.8–12 × 0.5–8 cm, chartaceous, moderately to densely pubescent adaxially and abaxially, sand punctate abaxially, the rachis densely pubescent, the margins entire to irregularly revolute resulting in somewhat undulate margins, the leaflets decreasing markedly in size toward the base of the leaf, the distal leaflet of the lowermost pair typically smaller than its match or completely absent; interjected leaflets absent; lateral leaflets 0.3–3.5 × 0.2–1.5 cm, ovate to elliptic, the bases rounded to truncate, oblique, the apices obtuse to acute, the petiolules nearly lacking to 2 mm long, moderately to densely pubescent; apical leaflet 1.2–8(–9.5) × 0.7–3(–4) cm, ovate to elliptic to oblong, the apex obtuse to acute to acuminate, the base obtuse to truncate to cordate, the petiolules nearly lacking to 7 mm long, moderately to densely pubescent; petioles 0.1–2.5(–4.5) cm, moderately to densely pubescent. Pseudostipules present at most nodes, one per node, 0.5–1.5 × 0.4–0.8 cm, obliquely ovate to elliptic, sometimes lunate, the apices obtuse to acute, the bases sometimes strongly lobed, oblique. Inflorescence 1.5–3.5 × 1–3 cm, extra-axillary on main stems or terminal on short, axillary spur shoots, simple to sometimes once branched in the extra-axillary inflorescences, with 1–8 flowers (1–3 on spur shoots [mean= 1.9], 3–8 on main stems [mean = 4.6]), with all flowers apparently perfect, the axes densely pubescent; peduncle 0.5–1 cm long; rachis nearly lacking to 1.5 cm; pedicels 7–15 mm in flower, 10–20 mm in fruit, somewhat expanded distally in flower and fruit, spaced contiguously to 6 mm apart, articulated at the base. Spur shoots 0.5–3.5(–8) cm long, bracteate, with 2–8 bracts per shoot, the bracts similar in shape to the cauline leaves, simple to occasionally 3–5-pinnate, 1–15 mm long, with minute pseudostipules. Calyx 4–5 mm long, the tube 1–2.5 mm long, the lobes 2.5–3.5 × ca. 1.5 mm, ovate-lanceolate to oblong, acute at tips, moderately pubescent, sand punctate; fruiting calyx slightly accrescent, the lobes 4–4.5 × 1.5–2 mm, ovate-lanceolate to oblong. Corolla 0.8–1.5 cm in diameter, 2–6 mm long, pentagonal, white to violet, sometimes with yellow at tips or along the midveins of lobes, flat to strongly reflexed at anthesis, the lobes 1.5–5 × 3–5 mm, acute at apices, glabrous adaxially, moderately to densely pubescent abaxially along midvein of lobes, the trichomes becoming shorter toward the densely pubescent apices of the corolla lobes, the margins densely ciliate apically. Stamens equal, with filaments 0.5–1.5 mm long, nearly free to fused for about ½ their lengths, somewhat broadly flattened, nearly glabrous abaxially, densely pubescent adaxially and on margins; anthers 3–4.5 × 1–1.2 mm, oblong, incurved, connivent, yellow, the pores large, directed distally, opening into latrorse-introrse longitudinal slits with age. Ovary glabrous to sparsely pubescent; style 5–7 × 0.1–0.2 mm, exceeding stamens by 1.5–5 mm, cylindrical, glabrous to papillose in lower ½ to sparsely pubescent with long trichomes in the middle or in the lower ½; stigma capitate. Fruits 2–2.5 × 1.5–2 cm, ellipsoidal, rounded to very slightly obtusely pointed at apex, green with darker mottled striping when immature, orange when mature, glabrous to sparsely pubescent when young. Seeds 3–4.2 × 2–4 mm, flattened, lenticular, rounded to teardrop-shaped, with a 0.2–1 mm wide wing around the margins, the thickened part of the seed 1.8–2.2 × 1.5–2 mm, rounded to reniform, light to medium brown, the surface smooth, the wing yellowish-tan to transparent near the margins, with radial striations.

**Figure 1.**
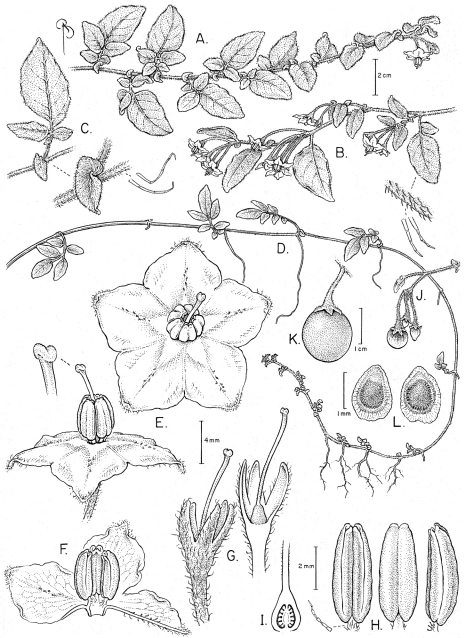
*Solanum baretiae* Tepe. **A** Habit of flowering branch **B** Flowering branch with close-up of pubescence **C** Pseudostipules with close-up of pubescence **D** Habit of vegetative branch **E** Flower and detail of stigma **F** Longitudinal section of flower **G** Calyx (left) and longitudinal section of calyx, showing ovary (right) **H** Stamens in ventral, dorsal, and side view with close-up of pubescence **I** Longitudinal section of ovary **J** Infructescence with immature fruits **K** Fruit, mature **L** Seeds. [**A** and **J** drawn from Tepe et al. 2888; **B–D, F–I** drawn from Tepe et al. 2886; **E** drawn from Tepe et al. 2885; **K–L** drawn from Bohs et al. 3735].

**Figure 2.**
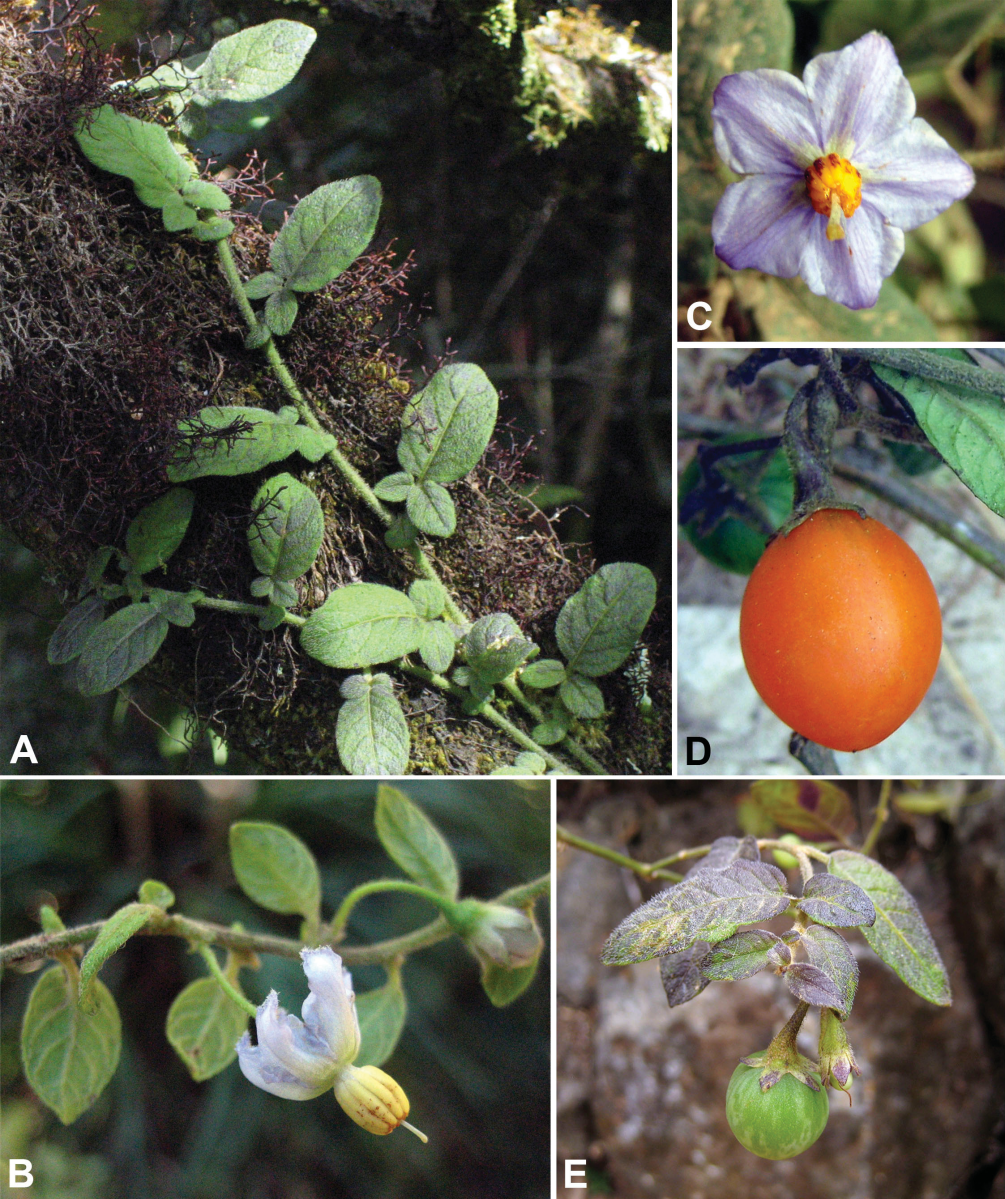
*Solanum baretiae* Tepe. **A** Habit **B** Flower showing reflexed corolla and bud **C** Flower with flat corolla **D** Mature fruit **E** Immature fruit; note mottling, which is absent in the mature fruit. [**A–B** Tepe and McCarthy 3346; **C** Tepe et al. 2885; **D** Bohs et al. 3735; **E** Tepe et al. 2888].

#### Distribution and ecology.

*Solanum baretiae* is apparently endemic to the Amotape-Huancabamba zone of southern Ecuador and northern Peru and grows in the understory of montane forests and disturbed roadside and pasture vegetation, 1900–3000 m in elevation. The areas where *Solanum baretiae* has been collected are seasonally dry.

#### Phenology.

Flowering specimens have been collected from Jun–Aug and Oct; fruiting specimens have been collected in May–Jun.

#### Etymology.

*Solanum baretiae* is named in honor of the botanist Jeanne Baret, the first woman to circumnavigate the earth (see below).

#### Preliminary conservation status.

According to the IUCN Red List Categories (IUCN 2011), *Solanum baretiae* is classified as Data Deficient (DD). Although *Solanum baretiae* occurs over a broad geographic range (> 60,000 km^2^), it has been collected at fewer than 10 localities (localities within a few kilometers of each other have been grouped for this assessment) and from a narrow elevational band within its range. The relatively small number of collections of this species suggests that it is rare in the habitats where it occurs. Furthermore, these localities are near expanding population centers and habitats in these areas are highly fragmented and degraded. Nevertheless, *Solanum baretiae* seems to be well suited to habitat change caused by human activities, since EJT and LB observed thriving populations along roadsides and among shrubs between the town of Guzmango (Dept. Cajamarca, Peru) and the cultivated and pasture lands that surround the town. Further data regarding the distribution and abundance of *Solanum baretiae* are needed before we can make a more solid assessment of its conservation status.

#### Specimens examined.

**ECUADOR. Loja:** 15 km S of Yangana, 4°25.43'S, 79°8.78'W, 2450 m, 31 Jul 2011 (fl), *E.J. Tepe and M. McCarthy 3346* (BM, MU, NY, QCNE, UT); Gualel, 3°43.5'S, 79°23.0'W, 2900 m, 10 Jun 1995 (fl, fr), *V. van den Eynden & E. Cueva 433* (NY). **PERU. Cajamarca:** Prov. Contumazá, Guzmango, 7°23.12'S, 78°53.73'W, 2600 m, 6 Jun 2010 (fr), *L. Bohs et al. 3735* (photos only); Prov. San Miguel, Miravalles Alto, Bolívar, 2600 m, 25 Aug 1991 (fl), *Solanum Llatas Quiroz 3021* (NY); Prov. Contumazá, alrededores de Guzmango, 2600 m, 27 Jul 1973 (fl), *A. Sagástegui A. 7711* (HUT, NY); Prov. Cajamarca, Namora–Matra, 2600 m, 16 Aug 1973 (fl), *A. Sagástegui A. 7751* (NY); Prov. San Miguel, entre Calquis y Llapa, 2400 m, 13 May 1977, *A. Sagástegui A. et al. 8863* (HUT, MO, NY); Prov. Contumazá, Contumazá–Ascabamba, 2700 m, 12 Jun 1981 (fl), *A. Sagástegui A. et al. 9991* (MO, NY); Prov. Contumazá, Santiago, 2450 m, 13 Jun 1983 (fl), *A. Sagástegui A. & S. López 10606* (BM, F, MO, NY); Prov. Contumazá, entrada al Bosque Cachil, 2500 m, 29 Jul 1993 (fl), *A. Sagástegui A. et al. 14982* (HUT, MO, NY); Prov. Contumazá, Bosque Cachil, 7°24.38'S, 78°46.88'W, 2500 m, 17 Oct 2010 (st), *E.J. Tepe et al. 2882* (HAO, USM, UT); Prov. Contumazá, ca. 5 km S of tunnel on Contumazá–Bosque Cachil road, , 2625 m, 17 Oct 2010 (st), *E.J. Tepe et al. 2884* (HAO, USM, UT); Prov. Contumazá, ca. 5 km S of tunnel on Contumazá–Bosque Cachil road, 7°24.33'S, 78°46.88'W, 2625 m, 17 Oct 2010 (fl), *E.J. Tepe et al. 2885* (BM, HAO, NY, PLAT, USM, UT); Prov. Contumazá, Contumazá–Guzmango road, 7°22.62'S, 78°53.63'W, 2850 m, 18 Oct 2010 (fl), *E.J. Tepe et al. 2886* (BM, HAO, NY, PLAT, USM, UT); Prov. Contumazá, Guzmango, 7°23.12'S, 78°53.73'W, 2600 m, 18 Oct 2010 (fl, fr), *E.J. Tepe et al. 2888* (BM, CINC, HAO, NY, USM, UT). **Lambayeque:** Prov. Ferreñafe, Bosque de Chiñama, 2300–2700 m, 15 Aug 1988 (fl), *A. Cano 2125* (NY); Prov. Lambayeque, Abra la Porculla, road from Olmos–Pucará, km 45 E of Olmos, 1920 m, 13 Jul 1986 (fl), *T. Plowman et al. 14284* (NY). **La Libertad:** Prov. Otuzco: abajo de Shitahoura (oeste de Salpo), 3000 m, 11 Jun 1992 (fl), *Solanum Leiva & P. Leiva 582* (NY); Prov. Otuzco: alrededores de San Andrés, 2560 m, 1 Jul 1992 (fl), *Solanum Leiva & J. Ullilen 646* (MO).

#### Discussion.

*Solanum baretiae* is a striking species with its relatively large, pentagonal corollas in shades of violet, yellow, or white (Fig. 2B), and its soft-pubescent leaves that range from simple to 7-foliolate. Specimens of *Solanum baretiae* have been previously identified as the Ecuadorian *Solanum chimborazense* Bitter, from which it differs by its larger corollas (0.8–1.5 cm in *Solanum baretiae* vs. < 1 cm in diameter in *Solanum chimborazense*), styles that are papillose or only sparsely pubescent (vs. densely pubescent with long trichomes in *Solanum chimborazense*), more flowers per inflorescence (1–8 in *Solanum baretiae* vs. mostly 1, but up to 3 in *Solanum chimborazense*), and filaments that are pubescent adaxially, but glabrous abaxially (vs. evenly pubescent on all surfaces in *Solanum chimborazense*). *Solanum baretiae* is sympatric with the exceedingly rare *Solanum chachapoyasense* Bitter but the latter species has stellate corollas (vs. pentagonal in *Solanum baretiae*), long filaments (3–3.5 mm in *Solanum chachapoyasense* vs. 0.5–1.5 mm in *Solanum baretiae*), and strictly simple leaves (vs. simple to 7-foliolate in *Solanum baretiae*). *Solanum baretiae* is also sympatric with several species of *Solanum* section *Basarthrum* (Bitter) Bitter, which can be scandent shrubs with compound leaves and somewhat similar flowers. These species, however, can easily be differentiated by the distinctive two-celled “bayonet” trichomes that characterize *Solanum* section *Basarthrum* (Seithe and Anderson 1982).

The Andean species of *Solanum* sect. *Anarrhichomenum* are typically found in mid- to high-elevation cloud forest habitats that are moist throughout the year. *Solanum baretiae* appears to be an exception to this rule, however, as it occurs in forests and disturbed areas on the western slopes of the Andes which, in the latitudes of the Huancabamba-Amotape zone, experience a marked dry season.

As mentioned above, the number of leaflets in this species is highly variable, with the leaves ranging from simple to compound with seven leaflets. Seedlings and young vegetative shoots typically have compound leaves with five leaflets, whereas the number of leaflets on fertile shoots is much more variable. In general, the number of leaflets, along with the size of the lateral leaflets, decreases along the length of fertile shoots, and the leaves in the proximity of the flowers and fruits are, in many cases, simple or have only one or two tiny lateral leaflets. The number of leaflets is variable in many species of *Solanum* sect. *Anarrhichomenum*, but the range of variability seen in *Solanum baretiae* is shared only with that of *Solanum sodiroi* Bitter (Anderson et al. 1999).

This species in named in honor of Jeanne Baret (1740–1807), an unwitting explorer who risked life and limb for love of botany and, in doing so, became the first woman to circumnavigate the world (Ridley 2010).

Jeanne Baret sailed on the ship *L’Étoile* in 1766 and embarked on the first French circumnavigation of the globe under the command of Louis Antoine de Bougainville (1729–1811) as assistant to the botanist Philibert Commerson (1727–1773). Since French naval regulations prohibited women being on board ship, Baret disguised herself as a man to join the expedition, and continued to wear men’s clothes during her time on the ship. Baret was Commerson’s lover, but was also an accomplished botanist in her own right and evidence suggests that she made some of the expedition’s most notable collections, including the showiest, most enduring botanical specimen from the expedition: the vine that would be named in honor of its commander, *Bougainvillea* Comm. ex Juss.

Commerson and Baret (though uncredited) amassed over six thousand specimens that are incorporated into the French National Herbarium at the *Muséum National d’Histoire Naturelle*. In the course of the expedition and the years after its successful completion, over seventy species would be named in honor of Commerson using the specific epithet *commersonii*. Expedition records show that Commerson was frequently unable to collect specimens in the field because of his health issues (Vivès 1766-1769) and, at these times, Baret took the part of the expedition’s chief botanist. Yet, today, despite the important role she played, not a single species is named after her. Commerson’s notes reveal that he intended to name a Malagasy genus *Baretia* (MS 887 of the Commerson archive in the *Muséum National d’Histoire Naturelle*), but it was never published (the species concerned are now placed in *Turraea* of the Meliaceae). The fact that individual plants of this genus that Commerson collected with Baret have leaves that are highly variable in shape perhaps struck him as a neat reflection of the multi-faceted companion who united seemingly contradictory qualities (Monnier et al. 1993): a woman dressed as a man, a female botanist in a male-dominated field, and a working class woman who had traveled farther than most aristocrats. Given the importance of her work and the singular nature of her achievements, Baret has clearly made a sufficient contribution to the field to deserve a species named after her. Following Commerson’s example, we believe that this new species of *Solanum*,with its highly variable leaves, is a fitting tribute to Baret.

## Supplementary Material

XML Treatment for Solanum baretiae
